# Dural-Based Glioblastoma Recurrence: An Illustrative Case and Review of Literature

**DOI:** 10.7759/cureus.96104

**Published:** 2025-11-04

**Authors:** Stephen Jaffee, Trent Kite, Matthew Shepard, Alexander Yu

**Affiliations:** 1 Neurosurgery, Allegheny Health Network, Pittsburgh, USA

**Keywords:** case report, dural spread, glioblastoma, recurrence, tumor

## Abstract

Glioblastoma is a common primary central nervous system tumor with an expected median overall survival of 12 to 15 months. Radiographically, high-grade gliomas classically present as heterogeneous ring-enhancing lesions in the brain parenchyma with central necrosis on MRI. In rare cases, other peripheral tumors can also present with a meningeal attachment with evidence of a dural tail. Histologically, the tumor has malignant features such as atypical cells, nuclear hyperchromasia, increased mitotic figures, angiogenesis, and necrotic regions. We present a 49-year-old male with a history of a left frontotemporal glioblastoma isocitrate dehydrogenase (IDH) wild type and O6-methylguanine-DNA methyltransferase (MGMT) unmethylated (~ 95% of gross total). The patient subsequently underwent chemotherapy with temozolomide and external beam radiation therapy with 60 Gy over 30 fractions. The patient presented eight months post-index resection with recurrence and dural invasion. The pathology report demonstrated adherent dural components. The patient was subsequently placed on bevacizumab. There is a paucity of literature regarding dural-based spread of glioblastoma along the dura after an initial resection. Additionally, there is difficulty in differentiating these tumors from meningiomas, with resultant misdiagnosis and management. It is critical to inform readers of its presence and emphasize the importance of its consideration in the differential diagnosis of dural-based tumors.

## Introduction

Glioblastoma multiforme (GBM) is a common primary central nervous system tumor that accounts for approximately 45% of malignant brain tumors [1]. Patients with GBM have a poor prognosis and a median survival ranging from 14.6 to 21.1 months with standard first-line therapy [2,3]. Most GBMs can be radiographically identified by their characteristic butterfly pattern that involves the corpus callosum and typically spreads into the bilateral temporal and occipital lobes. Less commonly, GBMs exhibit different growth patterns, making a diagnosis more difficult. One such growth pattern is growth towards the periphery contacting the dura, colloquially referred to as a 'dural tail sign,' which represents the regions of meningeal contact that are usually associated with meningiomas [4]. Given the classic association of a dural tail with meningiomas, lesions demonstrating this characteristic radiographic sign are often misdiagnosed [5-7]. The implications of misdiagnosis most importantly include delays in treatment and the receipt of inappropriate care. 

While the dural tail sign was first described as specific to only meningiomas [4], recent reports have found the dural tail sign’s specificity to meningiomas is closer to 94% [8-10]. Non-meningiomatous lesions exhibiting a dural-tail sign include lymphomas, chordomas, dural-based metastases, hemangiopericytomas, schwannomas, pleomorphic xanthoastrocytomas, and rarely GBMs [8,11]. Therefore, it is necessary to consider that not all dural tail signs on imaging can be completely explained by meningiomatous pathology during diagnosis [11]. Patients misdiagnosed with meningiomas based only on the presence of a dural tail sign are susceptible to undergoing inappropriate management and inappropriate delays in care. 

There are a few case reports of dural-based GBM in the literature. The majority of such cases deal with GBMs masquerading as meningiomas by exhibiting a dural tail sign. Incorrect treatments and delays in care can be detrimental for aggressive pathologies like high-grade gliomas. We describe the rare case of a patient with a recurrent dural-based GBM, compare it to other cases via a brief literature review, and discuss the implications of misdiagnosis.

## Case presentation

**Figure 1:**
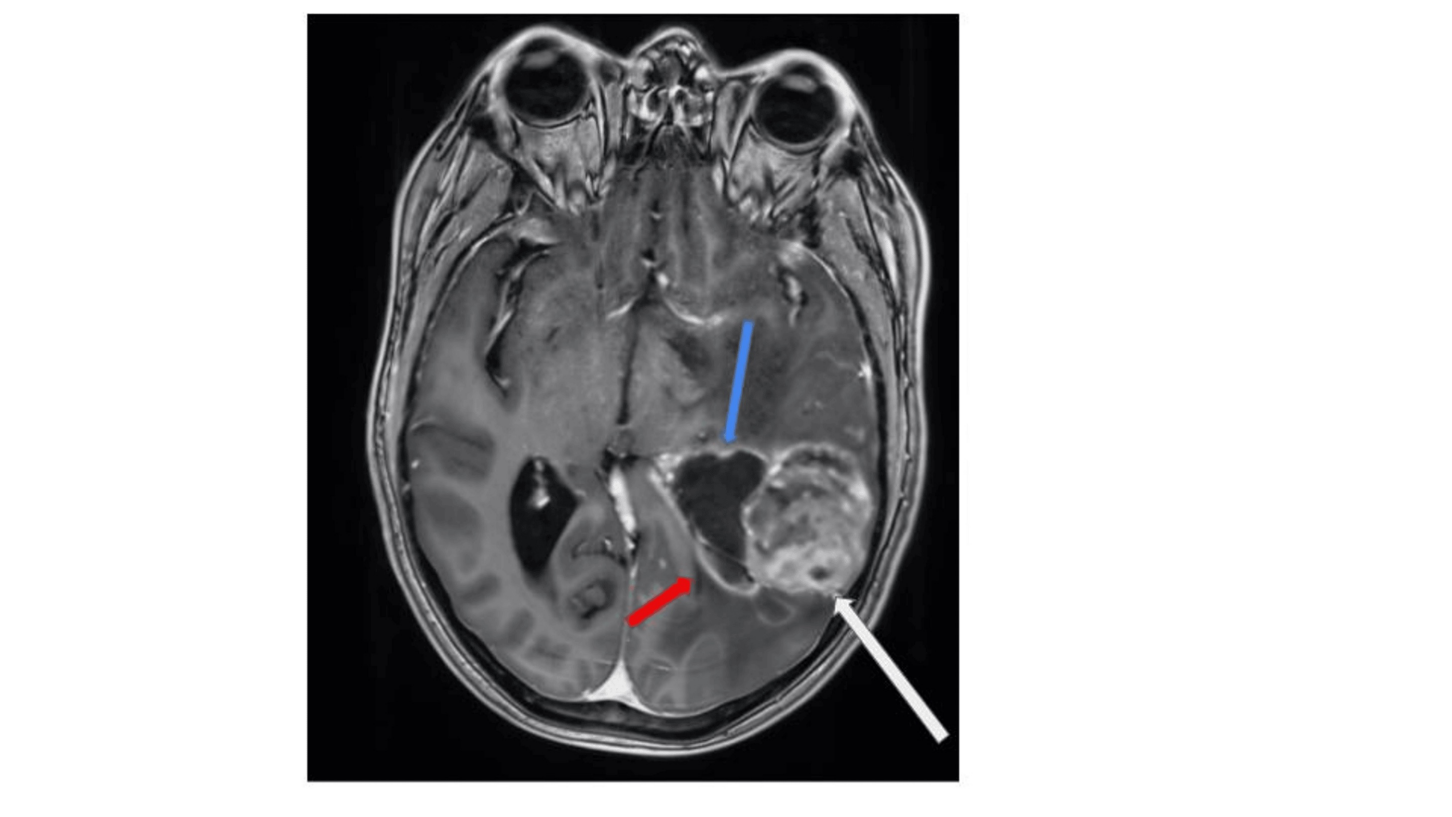
Preoperative MRI of the brain Initial preoperative MRI of the brain with and without contrast reveals a left posterior temporal heterogeneously enhancing lesion (white arrow) abutting the lateral ventricle (red arrow) with surrounding vasogenic edema (blue arrow).

The patient was a 49-year-old male with a medical history of acute lymphocytic leukemia status post chemotherapy (as a child). He presented to our institution with one week of altered mental status, including somnolence and aphasia. A CT scan of the patient’s head revealed a heterogeneous solid and cystic mass centered in the left frontoparietal region and significant vasogenic edema extending into the temporal lobe with mass effect causing partial effacement of the left lateral ventricle and approximately 9 mm of midline shift. The patient was started on levetiracetam for seizure prophylaxis and dexamethasone for cerebral edema. A CT of the patient’s chest, abdomen, and pelvis showed no potentially malignant lesions. An MRI of the patient’s brain demonstrated the lesion seen in Figure 1. 

**Figure 2:**
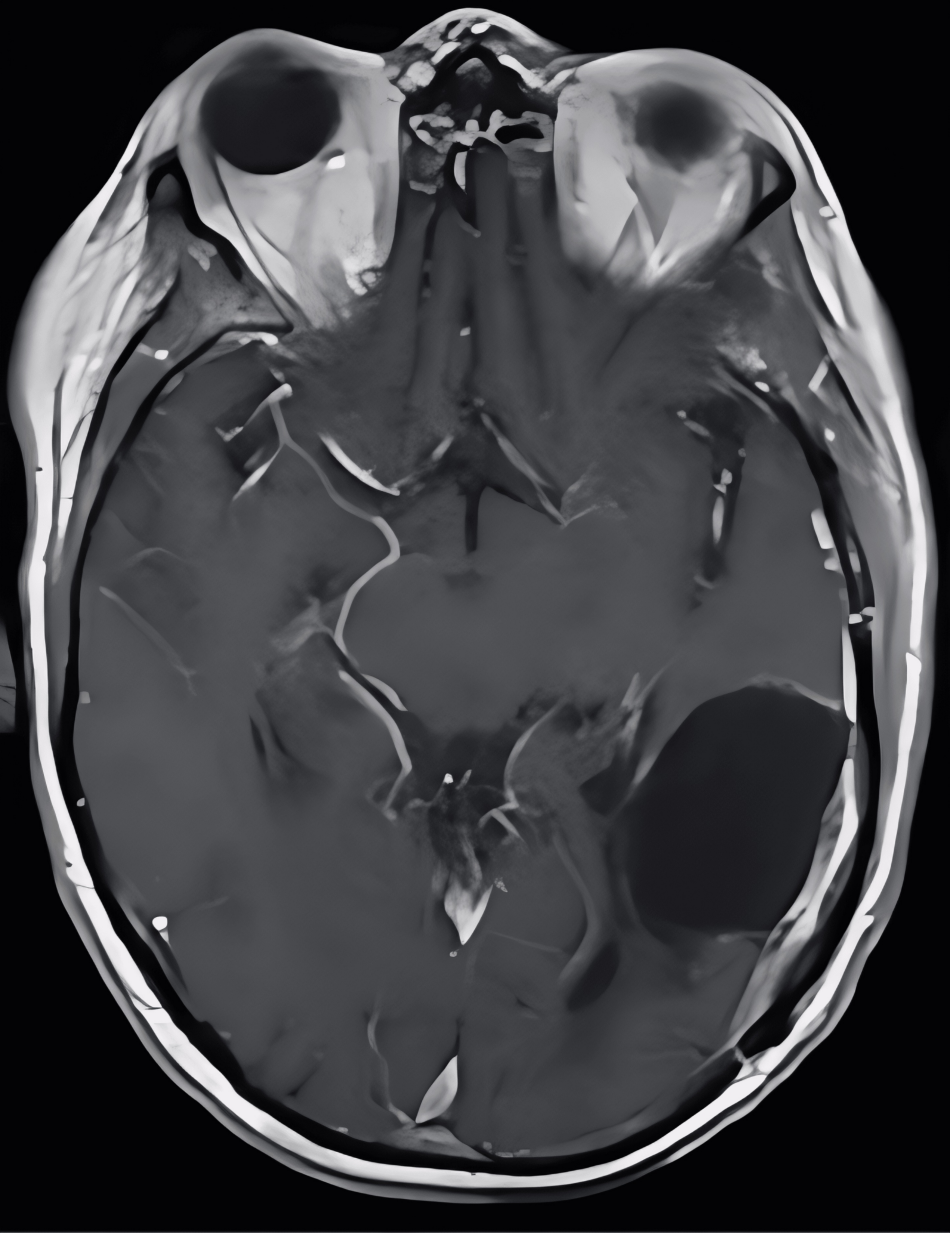
Postoperative MRI of the brain with and without contrast Initial postoperative MRI of the brain shows no significant residual tumor present.

**Figure 3:**
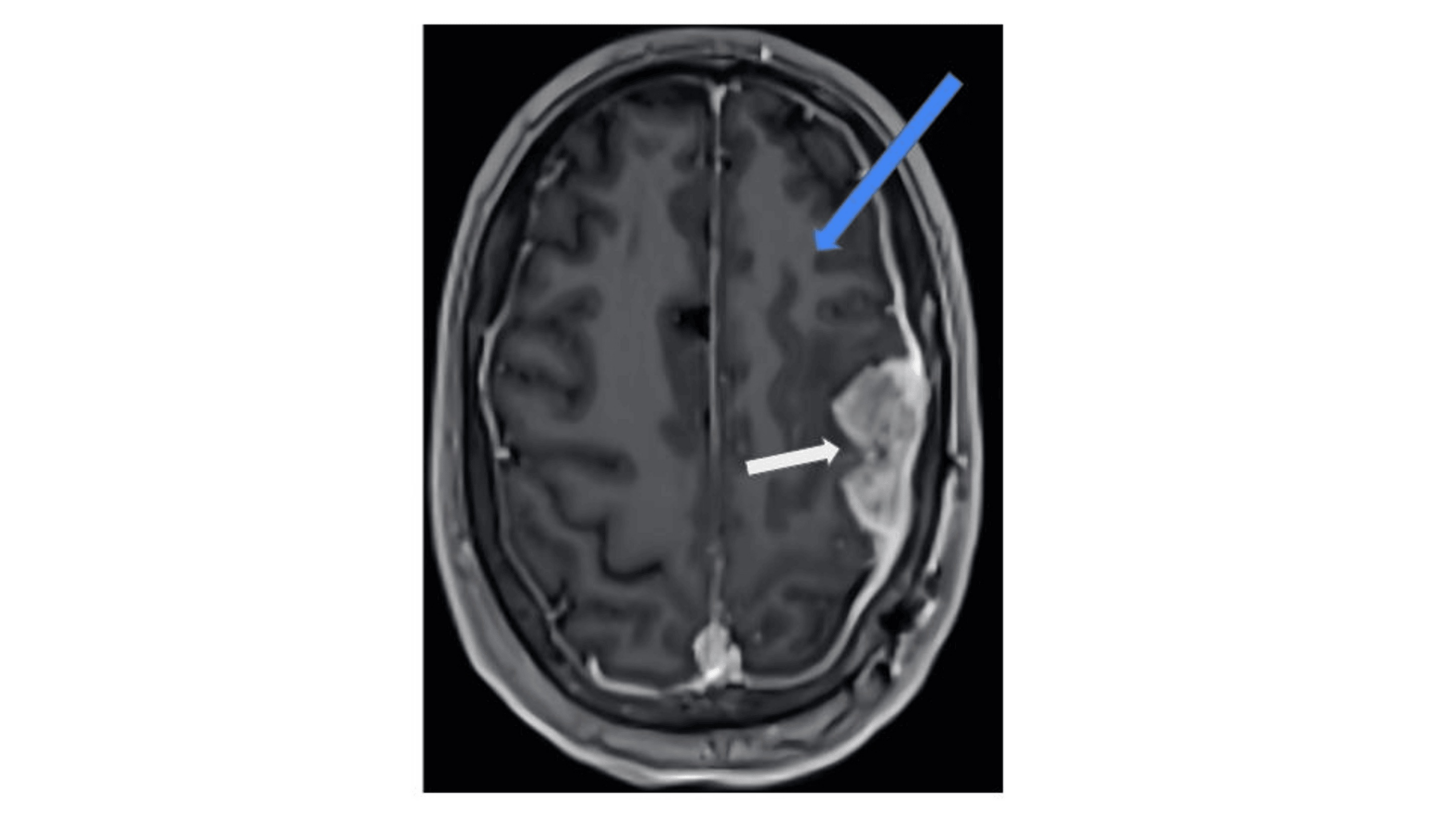
Interval MRI of the recurrent mass The interval MRI of the brain with and without contrast shows a heterogeneously enhancing lesion (white arrow) along the left-sided dural margin along the left frontal lobe (blue arrow).

The patient underwent a left temporoparietal craniotomy for brain tumor resection with complex duraplasty and cranioplasty (Figure 2). The patient had an uncomplicated postoperative course. An intraoperative pathology exam demonstrated a high-grade malignant neoplasm with necrosis, and the final pathology was found to be glioblastoma, isocitrate dehydrogenase 1 (IDH-1) (R132H)-wildtype, central nervous system (CNS) WHO Grade IV. The patient was discharged to inpatient rehabilitation on postoperative day seven with plans for adjunctive chemotherapy and radiation to the resection cavity under the care of medical and radiation oncology (standard 60 Gy over 30 fractions and concurrent chemotherapy with oral temozolomide at 75 mg/mL daily for 42 days during the radiation). At his one-month postoperative appointment, the patient’s confusion and aphasia symptoms were significantly improved. A follow-up MRI of the patient’s brain with and without contrast at eight months demonstrated post-surgical changes with a new dural-based lesion superior to the resection cavity (Figure 3).

**Figure 4:**
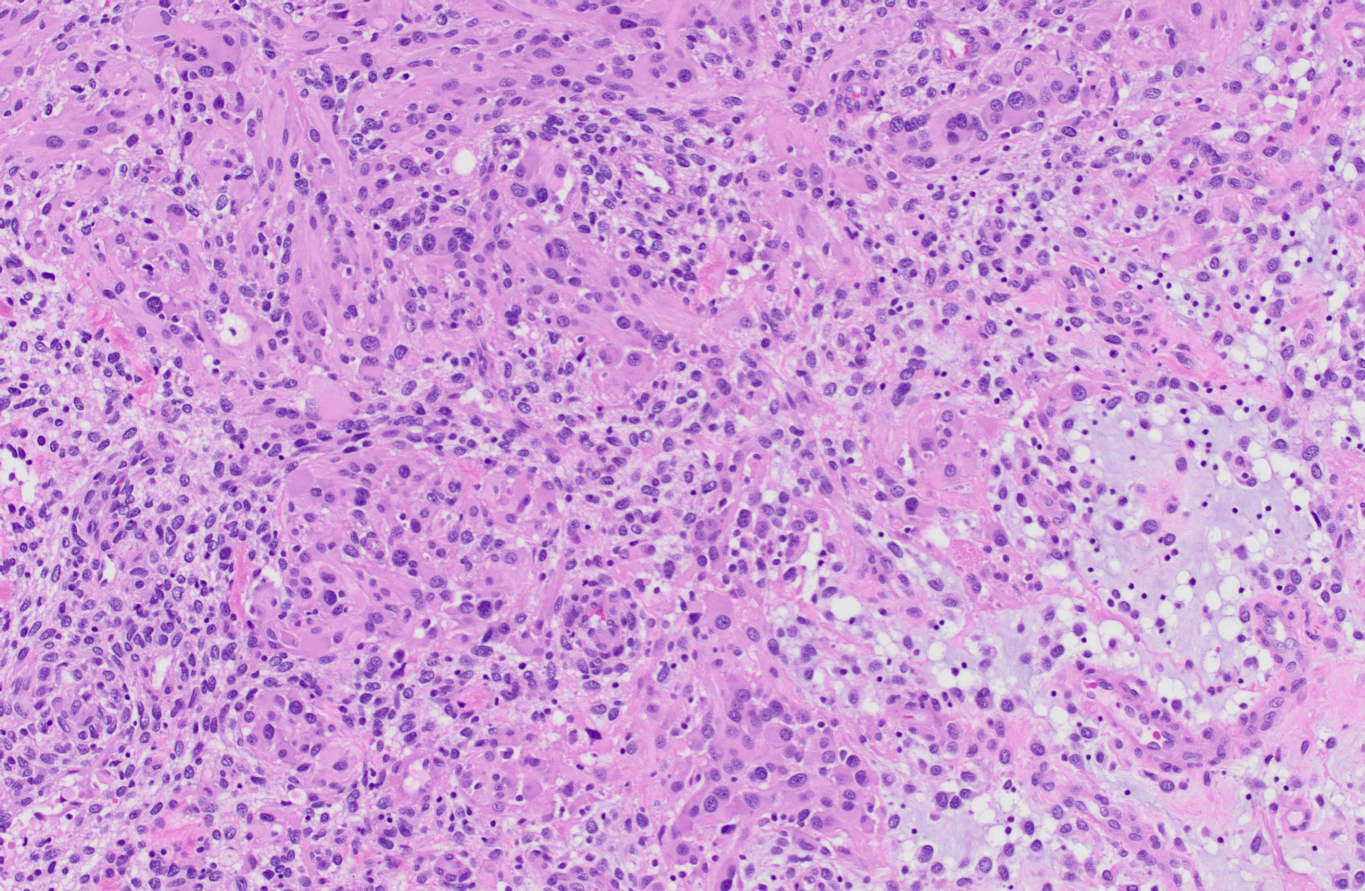
Primary lesion pathology The primary tumor was composed of epithelioid cells in a partially myxoid background on hematoxylin and eosin stain (magnification 100X)

**Figure 5:**
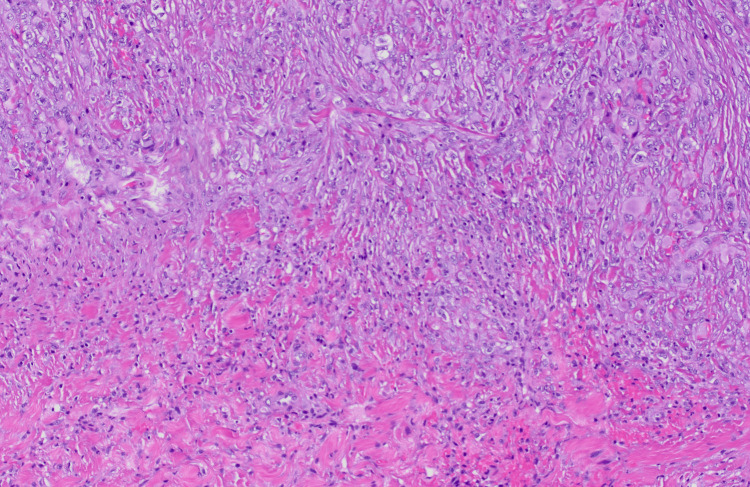
Pathology from recurrent lesion The recurrent tumor had a similar overall appearance and invaded the adjacent dura on hematoxylin and eosin stain (magnification 100X)

Over the next month, the patient began to develop worsening aphasia and displayed recurrent episodes of seizure-like activity, including intermittent right facial droop and right upper extremity weakness. Due to these new radiographic findings and the patient’s recent change in clinical status, the patient was offered another craniotomy for dural-based lesion resection and complex cranioplasty with plastic surgery assistance. The dural masses were resected en bloc with a dural margin; the lesion was also adherent to the temporal cortex; however, the lesion was successfully dissected away from the temporal cortex without any obvious temporal cortex resection. A dural repair was completed with a dural graft. The repeat pathology found multiple pieces of a tan-white-yellow and firm lobulated mass with adherent dura, measuring 7.0 x 5.0 x 1.0 cm. Final pathology revealed glioblastoma (IDH-1) wildtype, O6-methylguanine-DNA-methyltransferase (MGMT) unmethylated CNS WHO Grade IV tumor. Original pathology slides of the original and recurrent tumor are featured in Figures 4-5. The patient was subsequently placed on bevacizumab and was discharged with no postoperative complications. 

## Discussion

Our case demonstrated an atypical presentation of a recurrent WHO grade IV (IDH-1) wild-type (WT), MGMT unmethylated GBM presenting with a dural growth pattern, highlighting a need for caution when pursuing a diagnosis. Glioblastoma multiforme with dural involvement is rare, but the dural tail sign cannot be presumed to indicate meningiomas [12-15]. The importance of considering a GBM diagnosis is highlighted by Patel et al., where his two patients underwent pre-surgical embolization under the presumptive diagnosis of meningioma [6]. 

We identified nine reports in the literature representing 10 patients diagnosed with dural-based GBM [5-8,11,12-15]. Including our study, there are 11 patients included for discussion. The median patient age was 60 years; eight (73%) patients were male, and three (27%) were female. There were nine (64%) lesions that presented with a dural tail sign. In 10 (91%) cases, the provisional or differential diagnosis was a meningioma or non-GBM tumor subtype. Four (36%) patients underwent maximal safe resection followed by concurrent radiation and temozolomide (TMZ), and two (18%) patients underwent middle meningeal artery (MMA) embolization before resection. Seven (64%) patients were alive at their last follow-up or at the time of publication. It should be noted that the follow-up period was as short as three months [7], and one study omitted follow-up details beyond stating the patient was receiving treatment [6]. Of the 11 cases, three (27%) experienced progression or recurrence, with a time to event ranging from three to 42 months [12,13], and four (36%) died [15]. A summary of the current literature is tabulated in Table 1. 

**Table 1:** Baseline patient and treatment characteristics across studies reporting dural-based glioblastoma EBRT: External beam radiation therapy, TMZ: Temozolomide, MMA: Middle meningeal artery, PVA: Permanent embolic agents, WBRT: Whole brain radiotherapy, PNET: Primitive neuroectodermal tumors, CPA: Cerebellopontine angle

Study	Patients	MRI findings	Initial diagnostic impression	Final pathology	Management	Outcome
Michaelson et al. [5]	One 63-year-old male	Parafalcine frontal lobe heterogeneously enhancing mass with central necrosis, with broad dural attachment	Meningioma	Grade IV astrocytoma	Maximal safe resection followed by external beam radiation therapy (EBRT) and TMZ	Stable disease at two and a half years
Patel et al. [6]	1. 57-year-old Female 2. 60-year-old male	1. Heterogeneously enhancing right temporo-parietal mass with broad contact along the right tentorium, CSF cleft sign, and dural tail sign; 2. Left parasagittal, heterogeneously enhancing mass abutting the falx with a dural tail sign	1.Meningioma; 2.Meningioma	1. Grade IV glioblastoma; 2. Grade IV glioblastoma	Permanent embolic agents (PVA) particle embolization of the right MMA with subsequent resection; 2. PVA particle embolization of the MMA followed by resection	Not recorded
Wen et al. [7]	One 74-year-old male	Heterogeneous enhancement in the left temporal lobe with dural tail sign	Atypical eningioma, hemangiopericytoma, angiosarcoma, hypervascular metastases	Grade IV glioblastoma	Maximal safe resection followed by EBRT and TMZ	Stable disease at three months
Dabboucy et al. [11]	One 76-year-old male	Left temporal tumor broadly abutting the dural surface at its anterior inferior and medial aspects, with heterogeneous enhancement surrounded by extensive vasogenic edema. The dura showed a thickened appearance and enhancement of the dural tail sign surrounding the middle cranial fossa.	Malignant meningioma vs. glioblastoma	Grade IV glioblastoma	Maximal safe resection followed by EBRT and TMZ	Alive at the sixth-month follow-up
Hsieh et al.[15]	One 85-year-old female	Heterogeneously enhanced tumor abutting the dura laterally with an enhanced dural tail around the tumor, with CSF cleft sign	Meningioma	Grade IV glioblastoma	Maximal safe resection with whole brain radiotherapy (WBRT) and TMZ	Death at three months following treatment
Doddamani et al. [8]	One 17-year-old male	Heterogeneous predominantly solid cystic lesion with a central hypointense core suggestive of necrosis, with heterogeneous enhancement and a positive dural tail sign	Meningioma	Grade IV glioblastoma with primitive neuroectodermal tumor (PNET) components	Maximal safe resection with WBRT and TMZ	Alive
Stavrinou et al. [13]	One 53-year-old man	Temporoparietal mass attached to the dura with dural tail sign	Meningeal glioblastoma vs. meningioma	Grade IV glioblastoma	Resection	Recurrence at 42 months
Lee et al. [6]	One 71-year-old woman	Lobulated mass in the left CPA	Meningioma	Grade IV glioblastoma	Maximal safe resection followed by concurrent EBRT and TMZ	Progression at three months with stable disease at one year
This study	One 49-year-old male	Recurrent tumor with dural-based enhancing lesion superior to a left fronto-parietal resection cavity	Recurrent glioma	Grade IV glioblastoma	En bloc resection with adjuvant bevacizumab	Alive

Understanding the origin and nature of the dural tail sign is important for both surgical planning and potentially improving tumor diagnosis. There are currently several theories under consideration. The first is that the dural tail is either a tumor extension or vascular tissue proliferation [7]. This is supported by cases where the dural tail primarily demonstrates hyperproliferative connective and vascular tissue versus tumor cells [7]. In previous pathological analyses of GBMs with a dural tail, the tissues in the primary mass possessed different histologic features when compared to the dural tail [7]. In a series of five dural-based GBMs by Wilms et al., none of the patients demonstrated tumor cell infiltration in the tail [4]. 

Other theories also have support. A second explanation for dural tail development postulates that the neuroglial cells of the pia and arachnoid in adjacent parenchyma are responsible for propagating tumor growth [8,13,16]. Alternatively, there is evidence that tumor growth occurs from the proximal aspect of cranial nerves [8,16]. In the current case, we demonstrated local spread after subtotal resection. Pathological analysis documented adherence of the GBM to the dural component, but no true invasion was observed, indicating spread along the dural plane instead of growth within the dura itself. Our case supports this third hypothesis. Lastly, it has been suggested that GBM often spreads via white matter tracts and utilizes direct infiltration of surrounding tissue. The disruption of normal anatomical planes during surgery could induce local seeding along the dural surface.

## Conclusions

While rare, glioblastoma has been shown to spread along dural margins in episodes of recurrence. Because this growth pattern is most seen in meningiomas, it can allow GBMs to masquerade as meningiomas and result in misdiagnosis and an unsuitable treatment plan. Consideration of this phenomenon is important when diagnosing and treating a dural-based lesion.
